# Treating epistaxis – who cares for a bleeding nose? A secondary data analysis of primary and secondary care

**DOI:** 10.1186/s12875-021-01411-1

**Published:** 2021-04-15

**Authors:** Annina E. Althaus, Jonas Lüske, Ulrike Arendt, Michael Dörks, Michael H. Freitag, Falk Hoffmann, Kathrin Jobski

**Affiliations:** 1 Division of General Practice and Family Medicine, Department of Health Services Research, Carl von Ossietzky University of Oldenburg, Oldenburg, Germany; 2 Carl von Ossietzky University of Oldenburg, Ammerländer Heerstraße 114-118, 26129 Oldenburg, Germany; 3 Department of Audiology and Phoniatrics, Charité - Universitätsmedizin Berlin, Berlin, Germany; 4 Division of Outpatient Care and Pharmacoepidemiology, Department of Health Services Research, Carl von Ossietzky University of Oldenburg, Oldenburg, Germany

**Keywords:** Epistaxis, Nosebleed, Outpatient management, GP-centered care, Economic impact, Allocation

## Abstract

**Background:**

The primary objective was to describe outpatient treatment of epistaxis among different physicians based on a large patient population over a period of 10 years. The secondary objective was to evaluate the value of the practice fee as an instrument of allocation in patients with epistaxis.

**Methods:**

Anonymized statutory health insurance data (AOK Lower Saxony) of patients with a diagnosis of epistaxis treated between 2007 and 2016 were examined. Demographic data, accompanying diagnoses, medication and involved medical groups (general practitioners (GP), pediatricians, ear, nose and throat (ENT) specialists or other) were analyzed. Furthermore, we assessed whether the use of specialist groups changed after abolition of the practice fee in 2013.

**Results:**

Epistaxis was responsible for 302,782 cases (160,963 patients). The distribution of cases was slightly in favor of ENT specialists vs. GP (119,170 vs. 110,352). The cases seen by GP and ENT specialists were comparable with regard to age and sex distribution. Hypertension, atrial fibrillation/flutter and an antithrombotic therapy were slightly more common among cases consulting a GP. The GP recorded more co-diagnoses than the ENT. The use of outpatient care and the proportions of the involved physicians scarcely fluctuated during the study period. Overall, 23,118 patients (14.4%) were diagnosed by both, GP and ENT during a relatively short time period. The practice fee remuneration had no impact on the consultation of the physician groups.

**Conclusion:**

The outpatient treatment of epistaxis constitutes a considerable medical and economic burden in Germany. Strengthening the primary medical sector (GP-centered care) is necessary to reach the goal of initially directing patients to primary care, providing specialists more time for severe cases and reducing the impact on public health balance sheets.

## Background

Epistaxis describes different forms of nasal blood loss and is a common symptom that occurs in medical practice. The lifetime prevalence of epistaxis is estimated to be 60%, but as only up to 10% of patients require contact with the health care system, the prevalence might be underestimated [1–4].

Cases of epistaxis vary from an easily controlled bleeding to a highly acute event with life-threatening blood loss. The majority of epistaxis originates from the area of the anterior septum [5, 6]. Bleeding in this respect can be stopped with a few simple methods - in about 65% of cases, compression of the anterior septum and application of decongestant nasal drops are sufficient [2].

In Germany, both, general practitioners (GP) and specialists are involved in the outpatient care of patients. Patients with epistaxis may be treated by GP, pediatricians or ear, nose and throat (ENT) specialists, however, the consultation of an ENT specialist does not always seem to be necessary [1].

In the inpatient medical setting, care is provided by the emergency departments and the ENT departments of the hospital.

Germany has a dual public-private health care system financed by monthly statutory contributions (statutory health insurance (SHI)) and top up state cover (private health insurance), if requested. Germany has one of the highest healthcare expenditure relative to the gross domestic product (GDP) in Europe [7]. The contributions cover most of the costs. Due to the free choice of physicians (formal gatekeeping is missing), the number of consultations per patient in Germany is very high in an international comparison [3, 4]. With the introduction of the “practice fee” (10 euros per 3 months (quarter) of a year), which those insured by the SHI had to pay once a quarter when visiting a physician, an attempt was made to reduce the high consultation rates.

In addition to cost savings, the practice fee aimed to strengthen the function of the GP as the first point of contact. After an initial decline in patient contacts in the first years after its introduction in 2004, no sustainable effect could be demonstrated in the following years [8, 9]. The practice fee was abolished on 1 January 2013.

### Aim of the study

As there is little data on outpatient treatment of patients with epistaxis in Germany [10], we aimed to assess its frequency of occurrence, the use of outpatient care and its distribution among groups of physicians (e.g. GP or pediatrician versus ENT specialist) on the basis of a large patient population over a period of 10 years. Furthermore, we analyzed the value of the practice fee as an instrument for allocation in patients with epistaxis.

Finally, taking epistaxis as a model disease - high occurrence rate and affection of all age groups – we wanted to assess the efficiency of primary care.

## Methods

### Underlying data and case definition

The study was based on SHI data of the years 2007–2016 from the Allgemeine Ortskrankenkasse (AOK) Lower Saxony. In 2016, 2.5 million persons were insured with the AOK Lower Saxony, representing around 36% of all those insured by the SHI in the German federal state of Lower Saxony [11].

The study population comprised all insured persons who had at least one outpatient diagnosis of epistaxis between 2007 and 2016. Consequently, all outpatient cases of epistaxis (R04.0 according to ICD-10, German modification, diagnostic certainty “secured”) were included along with the specialty of the diagnosing physician (GP, ENT, pediatrician or another specialist group).

Since outpatient diagnoses are reimbursed on a quarterly basis in Germany (i.e. four three-month periods per year), each case was allocated to a diagnosis quarter. Consultations with the same case number (referring to a specific patient, case, physician and quarter) were considered only once. Vice versa, a patient could become a case more than once if he or she received multiple epistaxis diagnoses by different physicians and / or in different quarters during the study period.

Further details on the database and results on differences between the outpatient and inpatient groups are published elsewhere [12].

### Diagnoses, medication and fee positions

For all epistaxis patients, the data provided demographic information such as age and sex. Further, all outpatient diagnoses were available for the respective quarter in which the epistaxis was diagnosed. Only diagnoses coded with the diagnostic certainty “secured” were considered. These diagnoses were examined on two levels: (i) we assessed predefined comorbidities coded in the same quarter as the epistaxis (i.e. recorded by all physicians consulted by the patient in the respective quarter), (ii) we determined predefined conditions and the number of different ICD codes (on the 5-digit level) recorded with the epistaxis (i.e. with the same case number as described above).

Antithrombotic agents (B01 according to the anatomical-therapeutic-chemical (ATC) code) prescribed and reimbursed by the SHI were available for all epistaxis cases. Based on the prescription date and the prescribed number of defined daily doses (DDD), an epistaxis case was considered being currently treated with antithrombotic agents if he or she had respective medication on at last 1 day of the quarter of the epistaxis diagnosis.

Lastly, we assessed predefined fee positions according to the uniform fee position regulation (EBM) coded with an epistaxis case (i.e. with the same case number).

### Statistical analysis

Analyses were first conducted on the case level. We characterized epistaxis cases by age, sex, diagnoses, medication and fee positions stratified by the specialty of the physician diagnosing the epistaxis. Cases were further displayed by the years of the epistaxis diagnoses.

Second, analyses were conducted on the patient level stratifying patients by the age of their first epistaxis diagnosis during the study period. We determined which diagnosing physician group was consulted at least once. We further assessed whether a patient consulted a GP and an ENT specialist (i) at least once during the study period or (ii) during one quarter or two consecutive quarters. Using the date of the abolition of the practice fee, we last displayed the number of patients consulting the respective physician groups at least once before and since 2013.

We displayed case and patient characteristics using descriptive statistics (median, interquartile range (IQR) and percentages).

The data analysis was performed with SAS (Version 9.4, SAS Institute Inc., Cary, NC, USA).

### Ethics

As the article used anonymized secondary data, patient-informed consent was not required by German regulations.

## Results

### Epistaxis cases: characteristics, treatment and involved medical specialists

A total of 302,782 outpatient epistaxis cases (overall 160,963 patients) were recorded between 2007 and 2016. Most cases were seen by ENT specialists and GPs, followed by pediatricians and other specialists. About 8% of cases could not be assigned to a physician specialty. With the exception of those treated by pediatricians, the cases seen by GPs and ENT specialists were comparable with regard to the age and sex distribution (52 versus 49 years and 54.1% versus 55.9% men; see Table 1).

**Table 1:** Characteristics of outpatient cases (*n* = 302,782)

	**GP** **(*n* = 110,352)**	**ENT specialist** **(*n* = 119,170)**	**Pediatrician** **(*n* = 28,986)**	**Other specialist** **(*n* = 20,298**)	**Total** (***n* = 302,782)**
Median age, years (IQR)	52 (21–74)	49 (17–72)	7 (4–11)	58 (23–75)	46 (16–72)
Sex					
Male	59,723 (54.1%)	66,618 (55.9%)	16,957 (58.5%)	10,338 (50.9%)	166,877 (55.1%)
Female	50,629 (45.9%)	52,552 (44.1%)	12,029 (41.5%)	9960 (49.1%)	135,905 (44.9%)
Comorbidities/ antithrombotic medication^a^					
Arterial hypertension (I10-I15)	50,642 (45.9%)	47,118 (39.5%)	369 (1.3%)	9960 (49.1%)	120,702 (39.9%)
Chron. ischemic heart disease/ Coronary heart disease (I25)	17,789 (16.1%)	17,305 (14.5%)	38 (0.1%)	3797 (18.7%)	43,970 (14.5%)
Atrial fibrillation/flutter (I48)	11,704 (10.6%)	10,667 (9.0%)	23 (0.1%)	2557 (12.6%)	28,857 (9.5%)
Antithrombotics	20,417 (18.5%)	19,855 (16.7%)	62 (0.2%)	4538 (22.4%)	51,508 (17.0%)
Co-diagnosed diseases^b^					
Acute					
Respirator. inf. (B34.9, J06, J98.7)	10,410 (9.4%)	2898 (2.4%)	8847 (30.5%)	1452 (7.2%)	24,000 (7.9%)
Acute rhinitis (J00)	1999 (1.8%)	2729 (2.3%)	1580 (5.5%)	347 (1.7%)	6751 (2.2%)
Acute bronchitis (J20)	4445 (4.0%)	187 (0.2%)	2543 (8.8%)	729 (3.6%)	7949 (2.6%)
Hypertensive urgencies (I10.91)	2072 (1.9%)	111 (0.1%)	6 (0.0%)	293 (1.4%)	3171 (1.1%)
Chronic					
Chron. rhinitis (J31.0)	3555 (3.2%)	26,301 (22.1%)	1678 (5.8%)	533 (2.6%)	32,127 (10.6%)
Chron. sinusitis (J32)	3176 (2.9%)	6391 (5.4%)	329 (1.1%)	450 (2.2%)	10,377 (3.4%)
Chron. bronchitis/ COPD (J44)	8642 (7.8%)	355 (0.3%)	374 (1.3%)	1309 (6.5%)	10,703 (3.5%)
Traumatic					
Nasal bone fracture (S02.2)	237 (0.2%)	478 (0.4%)	11 (0.0%)	63 (0.3%)	1046 (0.4%)
Nasal bone contusion (S00.3)	475 (0.4%)	365 (0.3%)	327 (1.1%)	82 (0.4%)	1749 (0.6%)
Foreign body, nose (T17.0, T17.1)	18 (0.0%)	662 (0.6%)	11 (0.0%)	11 (0.1%)	711 (0.2%)
Acquired deformity of the nose (M95.0)	9 (0.0%)	7011 (5.9%)	1 (0.0%)	128 (0.6%)	7.155 (2.4%)
Neoplastic					
Malignant tumor of the nasopharynx, nasal (adjacent) cavity (C11, C30, C31)	45 (0.0%)	101 (0.1%)	0 (0.0%)	6 (0.0%)	154 (0.1%)
Myelodysplastic syndrome (D46)	185 (0.2%)	9 (0.0%)	0 (0.0%)	58 (0.3%)	258 (0.1%)
Hematological					
Thrombocytopenia (D69.4–.6)	870 (0.8%)	125 (0.1%)	32 (0.1%)	186 (0.9%)	1235 (0.4%)
Hemophilia A and B (D66, D67)	99 (0.1%)	17 (0.0%)	20 (0.1%)	9 (0.0%)	151 (0.1%)
Willebrand-Jürgens Sy.(D68.0)	186 (0.2%)	83 (0.1%)	112 (0.4%)	105 (0.5%)	501 (0.2%)
Hereditary hemorrhagic telangiectasia (I78.0)	391 (0.4%)	268 (0.2%)	1 (0.0%)	67 (0.3%)	756 (0.3%)
Cirrhosis of the liver (K74)	717 (0.7%)	66 (0.1%)	4 (0.0%)	97 (0.5%)	892 (0.3%)
Liver failure (K70, K72)	700 (0.6%)	16 (0.0%)	1 (0.0%)	135 (0.7%)	854 (0.3%)
Structural					
Nasal septum deviation (J34.2)	1203 (1.1%)	12,357 (10.4%)	24 (0.1%)	243 (1.2%)	13,856 (4.6%)
M. Osler (I78)	481 (0.4%)	1514 (1.3%)	16 (0.1%)	95 (0.5%)	2135 (0.7%)
Sicca Syndrome (M35.0)	210 (0.2%)	46 (0.0%)	0 (0.0%)	19 (0.1%)	275 (0.1%)
Medications /Noxae					
Drug abuse (F55)	72 (0.1%)	56 (0.1%)	1 (0.0%)	10 (0.1%)	148 (0.1%)
Cocaine abuse (F14)	4 (0.0%)	1 (0.0%)	0 (0.0%)	11 (0.1%)	16 (0.0%)
Alcohol addiction (F10)	2297 (2.1%)	80 (0.1%)	1 (0.0%)	340 (1.7%)	2796 (0.9%)
Inflammatory					
Allergy (T78.4)	3502 (3.2%)	1136 (1.0%)	1064 (3.7%)	706 (3.5%)	6416 (2.1%)
Allergic rhinitis (J30)	5033 (4.6%)	7230 (6.1%)	2241 (7.7%)	712 (3.5%)	15,239 (5.0%)
No other diagnosis	9581 (8.7%)	33,493 (28.1%)	2928 (10.1%)	2799 (13.8%)	66,060 (21.8%)
Median number of co-diagnosed diseases (IQR)^b^/^c^	5 (2–12)	1 (0–3)	3 (1–5)	5 (2–11)	2 (1–6)

Note: ^a^Diagnosed in the same quarter as the epistaxis

^b^Diagnosed with the epistaxis (same case number)

^c^at 5-digit level of the ICD code. *GP* general practitioner, *ENT* ear, nose and throat, *IQR* interquartile range

With regard to the comorbidities recorded in the quarter of the epistaxis diagnosis, there were no differences between GP and ENT consultations, except for slightly more frequent diagnoses of arterial hypertension and atrial fibrillation/flutter (no testing for significance). Antithrombotic therapy was recorded in 20,417 cases (corresponding to 18.5%) seen by the GP and in 19,855 cases (corresponding to 16.7%) consulting an ENT specialist.

Respiratory infections were the most frequently co-diagnosed diseases (with the same case number) among GPs, pediatricians and other specialists. Chronic rhinitis and nasal septum deviation were most common among ENT specialists (22.1%). The latter recorded the highest proportion of traumatic and malignant tumors as co-diagnoses; however, absolute numbers were low. Hypertensive urgencies were far more often recorded by GPs than by ENT specialists (1.9 vs 0.1%).

The median number of co-diagnosed diseases recorded by the GP was higher than the records by ENT specialist (median 1 by the ENT specialist, 5 by the GP and 3 by the pediatrician). The ENT specialist did not record any other disease besides the epistaxis in around 30% of the cases.

Table 2 shows the fee positions related to epistaxis. They are restricted to ENT specialist treatment only and not open for GP. It was found that a tamponade was only invoiced in 4.5% of all cases treated by the ENT specialist, while the additional fee position for the “treatment of acute, difficult to stop nose bleeding” was charged in 19.7% and the lupe laryngoscopy in 26.5% of all ENT-consultations due to epistaxis. Minor surgical interventions were invoiced in 12.1% (09360) and 13.7% (09361), respectively. The fee position of “minor surgical intervention in infants, toddlers and children” was invoiced in 1.2% of all epistaxis cases.

**Table 2:** Fee positions invoiced by ENT specialists according to the uniform fee position regulation (*n* = 302,782)

	**ENT specialist** **(*n* = 119,170)**
Tamponade of the posterior nasal sections and/or the nasopharynx (09310)	5308 (4.5%)
Lupe laryngoscopy (09311)	31,614 (26.5%)
Direct laryngoscopy using an endoscope in newborns, infants, toddlers or children up to 5 years of age (09313)	337 (0.3%)
Additional fee code for the treatment of a patient with acute, difficult-to-stop nosebleed (09329)	23,436 (19.7%)
Minor surgical intervention I in the ENT-mouth area (09360)	14,429 (12.1%)
Minor surgical intervention II in the ENT-mouth area and/or primary wound care in the ENT-mouth area (09361)	16,358 (13.7%)
Minor surgical intervention III in the ENT-mouth area and/or primary wound care in infants, toddlers and children in the ENT-mouth area (09362)	1461 (1.2%)

Note: Fee position 09329 is not to be requested on the same day as 09310 in a case. *ENT* ear, nose and throat

**Fig. 1:**
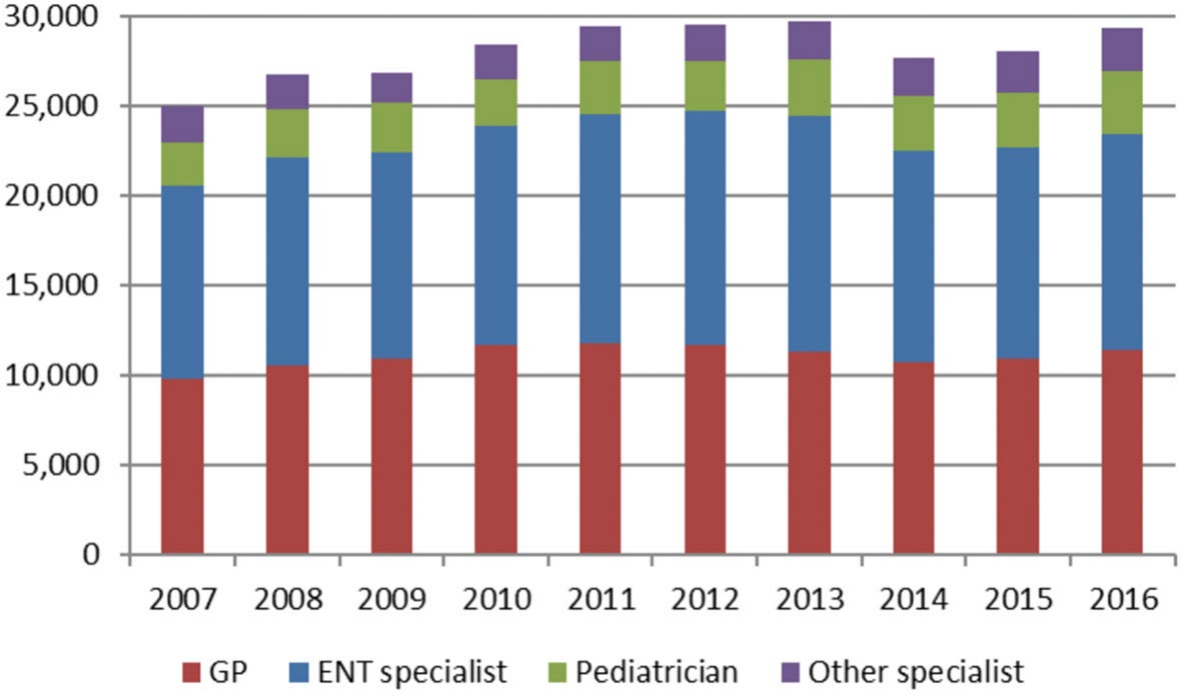
Epistaxis cases by year and place of diagnosis (*n* = 302,782)

Figure 1 shows the use of outpatient care and its distribution among the various specialist groups during the years 2007–2016. The proportion of GPs recording an epistaxis ranged from 38.1% in 2013 to 41.0% in 2010, whereas ENT consultations varied between 41.1% (2016) and 44.2% (2013).

### Patients with at least one epistaxis diagnosis during the study period: number of diagnoses, involved medical specialists and impact of practice fee

On the patient level, 98,351 of the 160,963 persons (61.1%), had only one epistaxis diagnosis during the study period. Of those patients with multiple diagnoses, nearly 30% were diagnosed more than once during one quarter or two consecutive quarters (data not shown). Overall, 23,118 patients (14.4%) received epistaxis diagnoses from a GP and an ENT specialist during one quarter or two consecutive quarters (Table 3).

**Table 3:** Number of medical specialists consulted at least once by patients’ age at initial diagnosis (*n* = 160,963)

	**0–10 years (*n*** **= 29,733)**	**11–20 years (*n* = 29, 346)**	**21–40 years (*n* = 24, 001)**	**41–60 years (*n* = 25, 017)**	**61–80 years (*n* = 38,275)**	**> 80 years (*n* = 14, 591)**	**Total (*n* = 160, 963)**
**Medical specialist(s) consulted at least once during the study period**^a^							
**GP**	6299 (21.2%)	14,190 (48.4%)	13,190 (55.0%)	13,030 (52.1%)	19,578 (51.2%)	7993 (54.8%)	74,280 (46.1%)
ENT specialist	11,803 (39.7%)	14,596 (49.7%)	11,900 (49.6%)	14,232 (56.9%)	23,412 (61.2%)	7477 (51.2%)	83,420 (51.8%)
Pediatrician	16,713 (56.2%)	4946 (16.9%)	27 (0.1%)	26 (0.1%)	54 (0.1%)	35 (0.2%)	21,801 (13.5%)
Other specialist	1853 (6.2%)	2098 (7.1%)	2188 (9.1%)	2513 (10.0%)	4470 (11.7%)	1770 (12.1%)	14,892 (9.3%)
**GP and ENT specialist consulted at least once**							
During the study period	2126 (7.2%)	4839 (16.5%)	3549 (14.8%)	4766 (19.1%)	8855 (23.1%)	3015 (20.7%)	27,150 (16.9%)
During one quarter or two consecutive quarters	1370 (4.6%)	3855 (13.1%)	3034 (12.6%)	4270 (17.1%)	7873 (20.6%)	2716 (18.6%)	23,118 (14.4%)

Note ^a^Patients might have consulted different physician specialties during the study period and, therefore, proportions sum up to over 100%

Looking at the distribution of epistaxis cases according to the patient’s age at first diagnosis of epistaxis, the youngest patients (0–10 years) consulted most often a pediatrician. From the age of 11 years on, the frequency of visits to the GP and ENT specialist was almost equal. The older patient population (61–80 years) consulted the ENT specialist most frequently (see Table 3). The proportions of the remaining group of other specialists ranged between 6 and 12%.

As illustrated by Table 4, during the practice fee remuneration (10 Euro per quarter in the years before 2013) there were only minor differences in the use of specialist groups compared to the years following its abolition.

**Table 4:** Patients who were diagnosed with epistaxis at least once before and after abolition of the practice fee in 2013 (*n* = 160,963)

	**2007–2012**	**2013–2016**	**Total**
	**(*n*** **= 100, 569)**^**a**^	(***n*** **= 74, 932)**^a^	**(*n*** **= 160, 963)**
**GP/Pediatrician**	58,632 (58.3%)	41,561 (55.5%)	94,486 (58.7%)
**ENT specialist**	52,587 (52.3%)	36,987 (49.4%)	83,420 (51.8%)
**Other specialist**	8649 (8.6%)	6727 (9.0%)	14,892 (9.3%)

Note: Patients might have consulted different physician specialties during one time period and, therefore, proportions sum up to over 100%

^a^Patients might have been diagnosed with epistaxis in both time periods

## Discussion

### Summary

Epistaxis was responsible for 302,782 cases corresponding to 160,963 persons insured with the AOK Lower Saxony. The cases seen by GP and ENT specialists were comparable with regard to age and sex distribution. Hypertension, atrial fibrillation/flutter and an antithrombotic therapy were slightly more common among cases consulting a GP. The GP recorded more co-diagnoses than the ENT.

The use of outpatient care and its distribution among the groups of physicians fluctuated scarcely between 2007 and 2016. Twenty-three thousand one hundred eighteen patients (14.4%) had been diagnosed by both ENT and GP during a relatively short time period.

The practice fee remuneration had no impact on the consultation of the physician groups.

### Consideration regarding patient allocation

The present study shows that epistaxis is a common symptom and has a high and rising impact on the German health care system. In contrast, population-based data on the epidemiology of epistaxis is scarce. Most studies in literature were either limited to a hospital setting or to specific populations like infants [13–17]. Next to differences in health care systems, this hampers comparisons in an international context. In a recent study using the same data basis, the prevalence of epistaxis treated in the in- and outpatient setting increased from 8.6 (2007) to 9.3 (2016) per 1000 insured persons (+ 21%) [12].

The patient groups seen by GPs and ENT specialists did not substantially differ in their age structure and comorbidities. This indicates that specialist i.e. ENT medical treatment is not only limited to severe epistaxis. This observation is confirmed by the analysis of the invoiced fee positions. Thus, a missing allocation of patients, i.e. a lack of gatekeeping, can be documented during the entire observation period of 10 years. The allocation of a patient to a GP, ENT or emergency department based on the severity of disease is not always reasonable. A recent cross-sectional study in an out-of-hours primary care center in northwestern Germany showed a remarkable high proportion of younger patients with non-urgent complaints [18]. In addition to differences in the access to these facilities, the assessment of urgency and the role of the primary care physician also differs between the countries.

The practice fee did not prevent the patient population of this study from visiting a specialist, thus the practice fee was not a useful instrument for cost-effective patient allocation in our study population. Whether patients’ own co-payments sensibly control the use of medical services is controversially discussed [19, 20]. We assume that a deductible has a controlling effect if medical services are demanded more often than actually necessary. In case of epistaxis the patient can rarely estimate the actual amount and seriousness of blood loss and the controllability by the patient’s own co-payment is limited. Furthermore, it is likely that 10 euros do not constitute a significant burden for many people. In addition, the waiting times for an appointment with an ENT specialist in Germany are comparatively short [3, 21].

The influence of the SHI Care Strengthening Act, also known as the Appointment Service Act, could not be conclusively examined in this study due to its introduction at the end of the study period (16.07.2015). The proportion of ENT consultations remained relatively stable during 2016 (see Fig. 1).

### Consideration regarding patient care

With regard to the care epistaxis patients received in the present study, it is striking that from the age of 20 years onwards a similar number of patients were treated by GP and by ENT specialists. Why is the GP involved in only about 36% of the cases, when 90–95% of all anterior epistaxis cases are proven to be easily treatable [10, 22] and a specific epistaxis therapy was only billed by the ENT specialist in every fifth patient? We assume that most patients directly consulted the ENT specialist without visiting the GP first or at all.

The German Society for General and Family Medicine (DEGAM) defines the responsibility of the GP as the first medical contact and basic care provider for all patients with physical and mental health disorders in emergency, acute and long-term care as well as areas of prevention and rehabilitation [23].

The present study cannot provide a conclusive assessment of the quality of GP care. However, the small number of patients (14.4%) who visited an ENT specialist and a GP in the same or two consecutive quarters suggests that primary care was successful in most cases and/or a referral to an ENT specialist was only necessary in every seventh patient. In addition to being easier to reach and thus providing faster emergency care, GPs are usually more familiar with the patient. Hypertensive urgencies, for example, were far more often documented by GPs than by ENT specialists. This is interesting in the sense that high blood pressure can be the cause of epistaxis. Further, low blood pressure can be a sign of high blood loss. Whether these measurements were performed as an assessment of the patient’s condition in an emergency situation or as a search for possible causes of epistaxis cannot be derived from the data. The average (median) number of recorded co-diagnoses, which suggest causal components, was also substantially higher among GPs than among ENT specialists.

Only malignant tumors were mainly recorded by the ENT physician due to the diagnostic value of the endoscopy. However, these were rather rare diagnoses.

Regardless of the severity of epistaxis, the ENT specialists consider their medical therapy as optimum care [10, 24]. The reasons for this are the incorrect assessment of blood loss and thus the severity of epistaxis by non-specialist physicians – in this case GP and pediatricians – and the frequent lack of basic emergency care [25, 26]. The latter statements are based on studies published 1993 and 2005, respectively. To our knowledge, there are no more recent studies.

### Future impact and possible improvements

The outpatient treatment of epistaxis constitutes a considerable burden. Due to the demographic development with an increase of age-related diseases and the associated increase in multimorbidity, a further increase of epistaxis is to be expected [26, 27]. This is further aggravated by the reduction in the number of specialists, especially in rural areas [28]. Primary care provided by GPs is likely to be sufficient for most epistaxis cases. We recommend studies to examine and, if necessary, optimize the quality of primary care for epistaxis.

The practice fee did not lead to better patient allocation between primary and secondary care. Thus, GP-centred care might be necessary to reach the goal of directing patients to primary care first [29]. It better addresses an adequate allocation of resources, providing specialists more time to consult on serious cases.

By means of targeted performance management and controlled allocation of (expensive) diagnostics, a managed care model allows holistic care in the sense of “disease management” [30]. On the one hand, this will lead to an improvement in efficiency, quality and continuity of care, on the other hand to possible cost savings by avoiding unnecessary examinations [31].

Taking epistaxis as a model disease its low cost-effective treatment may be reflected in the treatment of other conditions. We recommend population-wide studies regarding possible cost savings in the treatment of common diseases in the primary care sector.

### Strengths and limitations of the study

The major strengths of this study are the large database and the long time period of 10 years.

However, the present study is limited by the nature of the data of a single statutory health insurance. It is known that insurances differ with respect e.g. to demographics, socio-economic status and morbidity, which limits the generalizability of the results [32].

Furthermore, it was not possible to determine whether the patients first visited the GP and then, e.g. due to a referral, the ENT specialist, since the diagnoses could only be assigned to a quarter, but not to a specific date within this quarter. Possible budgetary reasons (e.g. total time budget at the end of the quarter), which could have influenced the billing of the ENT physician, could not be excluded either. Finally, the role of institutions such as ambulatory out-of-office-hours services which were probably included in the 8% of cases not linked to a physician specialty, could not be further evaluated.

Nevertheless, we considered the collected data to be relevant to analyze the outpatient care of epistaxis in Germany.

## Conclusion

The outpatient treatment of epistaxis, based on the results of this study, constitutes a considerable medical and economic burden in Germany. The present study indicates that the use of resources in common diseases such as epistaxis can be optimized. Strengthening the primary medical sector (GP-centered care) is necessary to reach the goal of initially guiding patients to primary care and reduce the impact on public health balance sheets.

## Data Availability

The data that support the findings of this study are available from the AOK Lower Saxony but restrictions apply to the availability of these data, which were used under license for the current study and are not publicly available. Data are however available from the authors upon reasonable request and with permission of the AOK Lower Saxony.

## Abbreviations

AOK: Allgemeine Ortskrankenkasse
ATC: Anatomical- therapeutic- chemical
DDD: Defined daily doses
EBM: Uniform fee position regulation
ENT: Ear, nose and throat
GP: General practitioner
IQR: Interquartile range
SHI: Statutory health insurance
